# Novel magnetic resonance technique for characterizing mesoscale structure of trabecular bone

**DOI:** 10.1098/rsos.180563

**Published:** 2018-08-29

**Authors:** Chantal Nguyen, Kimberly J. Schlesinger, Timothy W. James, Kristin M. James, Robert L. Sah, Koichi Masuda, Jean M. Carlson

**Affiliations:** 1 Department of Physics, University of California, Santa Barbara, UC Santa Barbara, Santa Barbara, CA 93106-9530, USA; 2 BioProtonics, LLC, 3090 Old Calzada Rd, Santa Ynez, CA 93460, USA; 3 Department of Bioengineering, Jacobs School of Engineering, University of California San Diego, 9500 Gilman Dr, La Jolla, CA 92093, USA; 4 Department of Orthopaedic Surgery, University of California San Diego, 9500 Gilman Dr, La Jolla, CA 92093, USA; 5 Center for Musculoskeletal Research, Institute of Engineering in Medicine, University of California San Diego, 9500 Gilman Dr, La Jolla, CA 92093, USA

**Keywords:** magnetic resonance, osteoporosis, histomorphometry, trabecular bone

## Abstract

Osteoporosis, characterized by increased fracture risk and bone fragility, impacts millions of adults worldwide, but effective, non-invasive and easily accessible diagnostic tests of the disease remain elusive. We present a magnetic resonance (MR) technique that overcomes the motion limitations of traditional MR imaging to acquire high-resolution frequency-domain data to characterize the texture of biological tissues. This technique does not involve obtaining full two-dimensional or three-dimensional images, but can probe scales down to the order of 40 μm and in particular uncover structural information in trabecular bone. Using micro-computed tomography data of vertebral trabecular bone, we computationally validate this MR technique by simulating MR measurements of a ‘ratio metric’ determined from a few *k*-space values corresponding to trabecular thickness and spacing. We train a support vector machine classifier on ratio metric values determined from healthy and simulated osteoporotic bone data, which we use to accurately classify osteoporotic bone.

## 1. Introduction

Osteoporosis, a metabolic bone disease which leads to increased bone fragility and fracture propensity, affects millions worldwide and results in a significant economic toll. In the USA, osteoporosis affects approximately 10.2 million adults over age 50 [1], and in Europe, the number of patients with disability due to osteoporosis is comparable to that of heart disease and greater than that of cancer [2]. The annual costs of osteoporotic fractures have been estimated at 20 billion USD in the US and 30 billion USD in the European Union [3]. Osteoporosis is diagnosed by estimating bone mineral density (BMD), typically accomplished using dual-energy X-ray absorptiometry (DXA) or quantitative computed tomography (QCT). However, BMD is poorly correlated with fracture likelihood; there is considerable overlap in BMD between healthy individuals and fracture patients [4], and it has also been shown that BMD cannot fully explain the variance in strength-related properties of bone [4–6].

We introduce a magnetic resonance (MR) technology called μTexture for probing the texture of various biological tissues. μTexture overcomes the motion limitations of existing MR imaging (MRI) methods to acquire high-resolution data that can inform the detection and monitoring of disease. A vast number of diseases, such as hepatitis C, non-alcoholic fatty liver disease and pulmonary fibrosis, are linked to changes in tissue texture in the heart, liver and other organs [7–9]. In this paper, we focus on the case of trabecular bone, a porous bone tissue resembling a network of interconnected spindles chiefly found in the interior of the vertebrae and the ends of long bones such as the femur. Trabecular bone is known to exhibit structural damage and changes in anisotropy with the onset and progression of osteoporosis and its less severe form, osteopenia [4].

While micro-computed tomography (micro-CT) is the current standard for obtaining high-resolution images of bone and other tissues, the large amount of radiation involved prevents its clinical use, limiting its application to isolated samples or small animals [4]. By contrast, μTexture is designed to be implemented clinically for diagnosis and monitoring of disease. MRI does not involve ionizing radiation, but existing MR methods cannot achieve the resolution of micro-CT. In traditional MRI, measurements are made in the spatial frequency domain; the raw data matrix is referred to as *k*-space, which is Fourier transformed to obtain the final image. Rather than acquiring a two-dimensional (2D) image, μTexture finely samples one point of *k*-space at a time to obtain high-resolution data in the spatial frequency domain, at frequencies relevant to the texture of the targeted tissue. Hence, μTexture is not limited by patient motion as in traditional MRI, and can probe smaller length scales than existing MR methods.

In this paper, we simulate μTexture measurements on the trabecular bone tissue to determine acquisition parameters that will provide valuable diagnostic information related to the structure of the probed tissue. We furthermore computationally validate the diagnostic ability of μTexture in the case of osteoporosis by developing a ‘ratio metric’ for classification of healthy and diseased bone.

Bone is a hierarchical material that exhibits mechanisms of fracture resistance across multiple scales. At the macroscale, human bone consists of two types, the dense, shell-like cortical bone, and the web-like trabecular (or cancellous) bone. Trabecular bone is found mostly in the vertebrae and at the ends of long bones, encased by a cortical shell. At the mesoscale, the structure of trabecular bone resembles a highly porous network of struts and rods (trabeculae) that are individually on the order of tens of microns in thickness. This structure results in a lightweight material with high stiffness and strength which can tolerate large deformations [4]. At the sub-microscale, individual trabeculae are made up of mineralized collagen fibrils, the ‘building blocks’ of bone, which are made up of hydroxyapatite crystals embedded in a collagen matrix [10]. The micromechanics of these components have been shown to be predictive of overall bone stiffness [11,12].

Trabeculae erode and perforate with age and disease, leading to wider spaces between them. The accumulation of microcracks and breakage in ageing bone contributes to its fragility. At the same time, the bone is constantly remodelling itself; old bone is resorbed and replaced with new bone, but an imbalance between resorption and formation results in osteoporotic bone loss, characterized partly by diminished BMD [4]. However, while BMD can explain the variance in the mechanical strength of trabecular bone only up to about 70%, a combination of BMD and structural properties such as anisotropy can explain up to 90% [4–6,13,14].

Trabecular architecture is quantified with histomorphometry, the study of the shape and form of tissue, typically from analysis of high-resolution images. Commonly used histomorphometric parameters include trabecular thickness (Tb.Th), which quantifies the average thickness of the trabeculae; trabecular spacing (Tb.Sp), which quantifies the average width of the gaps between trabeculae; and trabecular number (Tb.N), which measures the average number of trabeculae per unit length [15]. Trabeculae erode and perforate with age and the onset of disease, resulting in a decreased Tb.Th; Tb.N decreases as well, resulting in an increased Tb.Sp [16,17]. Histomorphometric parameters can thus serve as informative diagnostic markers for the health and strength of trabecular bone. Values for Tb.Th and Tb.Sp are typically reported as an average value over a region; however, the thickness and spacing can be highly variable throughout a volume of bone. Measures of variability in Tb.Th and Tb.Sp, e.g. moments or other characteristic quantities of their distributions, may provide further diagnostic information. The anisotropy of the trabecular structure has also been shown to be predictive of bone mechanics [18].

Micro-CT is the imaging standard for histomorphometry, but high amounts of radiation involved place limitations on acquiring high-resolution images of *in vivo* human bone. The highest resolution obtainable for *in vivo* imaging of bone is accomplished with HR-pQCT (high-resolution peripheral QCT), developed for use on distal extremities in humans, which images at a resolution of approximately 80 μm. Furthermore, the best resolution of micro-MRI (high-resolution MRI) is about 30 μm for *ex vivo* samples where motion effects are not prohibitive [19]; applied *in vivo* to peripheral locations, resolution of approximately 140 μm has been achieved in imaging bone [20]. In comparison, trabecular thicknesses are roughly 100 μm on average and are lower for osteoporotic bone. Here, we use *ex vivo* 9-μm resolution micro-CT images of human bone to obtain ground-truth histomorphometric measurements, and we simulate diseased bone profiles by virtually eroding bone elements in these images.

While μTexture is an MR technique, it is not a procedure that is applied to existing MR images. Rather, it is a technique for obtaining frequency-space data using clinical MR equipment without acquiring full 2D or 3D images. In this paper, we first introduce and detail the μTexture technique for probing biological tissues by measuring the MR signal at specific spatial frequencies relevant to the tissue texture. We then conduct an *in silico* validation of μTexture by simulating μTexture measurements, using micro-CT data as ground truth, to obtain spatial frequency information associated with trabecular structure. We start by transforming high-resolution micro-CT images into spatial frequency data, and extract a subset of this data at frequencies specifically chosen to be relevant to the structure of trabecular bone. We use the simulated μTexture measurements to calculate a ratio metric, which we then use to train a classifier to distinguish between healthy bone and bone that has been artificially eroded to simulate osteopenia and osteoporosis. We apply this classifier to bone with osteoporotic characteristics to show that the ratio metric can be used to identify diseased bone, indicating that a full 2D image is not required to yield diagnostic information derived from bone architecture.

## 2. Magnetic resonance technique for probing biological texture

We have developed an MR technique known as μTexture [21], which allows for fast acquisition of MR data from *in vivo* biological tissues while overcoming most of the motion limitations of other commonly used diagnostic MRI techniques [22,23]. μTexture is able to resolve the texture of biological tissues at wavelengths down to less than 40 μm, or even smaller in conjunction with machine learning techniques, compared with the approximately 80 μm resolution of HR-pQCT or approximately 140 μm resolution of micro-MRI available for *in vivo* human clinical use. In contrast with typical MRI, which acquires data from all or most of *k*-space and takes the Fourier transform to obtain an image, μTexture probes *k*-space one point (or small region) at a time, acquiring a measure of signal magnitude versus *k*-value (frequency) at the desired points or regions in *k*-space for a selected volume of tissue. That is, μTexture focuses on obtaining frequency-domain data at specific frequencies relevant to the texture of the targeted tissue.

Patient motion severely affects traditional MRI at the resolution required to image the fine texture of biological tissues. Even when the patient holds their breath during imaging, cardiac pulsatile motion and twitching can cause blurring. Imaging at higher resolution lengthens the data acquisition time and worsens the motion-induced blurring. Furthermore, on top of the longer times required to image higher *k*-values (shorter wavelengths), signal strength weakens as *k* increases. Because μTexture trades acquisition of a full 2D image for a high-resolution profile at a few chosen *k*-space values, the acquisition time required to obtain relevant frequency-domain information about the tissue is vastly reduced. μTexture acquires measurements from one *k*-value on the time scale of milliseconds, small enough such that blurring due to patient motion is negligible.

**Figure 1.**
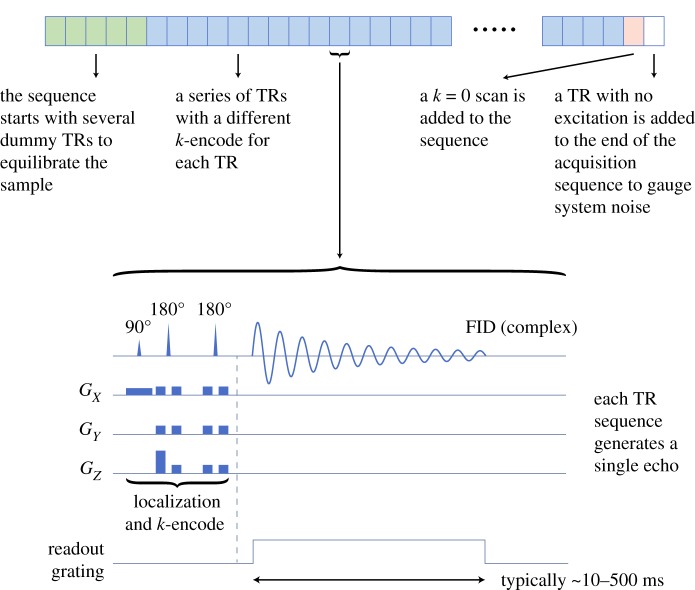
Example schematic of the μTexture measurement procedure, consisting of a repeated set of singular *k*-value encodings. Each TR encodes a different *k*-value; an example pulse sequence timing diagram for one TR is highlighted at the bottom of the figure. The free induction decay (FID) is acquired for the full T*_2_ period, from which the measure for a chemical species of interest (e.g. water) at the encoded *k*-value is determined. The dark blue rectangles in the timing diagram represent slice-select gradients, crusher gradients or *k*-encoding gradients. In this example, the *z*-axis is the analysis direction. Note that one of the crusher gradients is modified to *k*-encode the tissue.

μTexture uses a custom pulse sequence (figure 1) to isolate a small, targeted region, which is typically a prism with one dimension (1D) designated as the ‘analysis’ dimension and the other two the ‘cross-section’ dimensions. Within one μTexture excitation, the prism is excited and phase-encoded for the desired *k*-value or values (hereinafter referred to as a *k*-encode), and the signal is measured. Up to approximately 10, *k*-values can be measured in one repetition time (TR; the time interval between excitations), though figure 1 describes an example procedure in which one *k*-value is measured in each TR. These steps can be repeated several times within the same analysis volume and the magnitude of the signals can be averaged to improve the signal-to-noise ratio. The signals from several different non-overlapping prisms, in a technique called interleaved acquisition, can also be acquired in one TR. Additional wavelengths can be probed by repeating the encoding of other *k*-values over subsequent TRs, thereby building up a sampling of *k*-space pertinent to the pathogenesis of a disease.

As measurements from one *k*-value are done in a single TR, they are inherently immune to motion during signal recording. The protons in the volume of interest (VOI) are independent, without coherence or interference effects, and the proton spin direction is decoupled from the molecular orientation. The encoded spins move with the tissue regardless of translation, rotation or distortion of the tissue; as long as the VOI stays within the receiver and the homogeneous magnetic field, the signal is not affected. Furthermore, because μTexture probes texture, rather than acquiring an image, there is no need for precise spatial coherence between subsequent excited volumes. Hence, in a series of *k*-encodes over several TRs, each measurement is independent. Thus, μTexture is tolerant to motion across excitations, and this motion immunity is not tied to the fast (milliseconds-long) acquisition but to the fact that data within a chosen *k*-value are acquired within a single TR.

While in this paper we focus on probing trabecular bone through isolated vertebral samples that have been washed to remove soft tissue, μTexture can be used to measure multiple chemical species in a tissue that may have differing spatial compositions. Unlike typical MRI, μTexture can probe large enough regions with signal averaging to map chemical species as a function of wavelength. With volume selection and no *k*-encoding, μTexture can be used to measure the NMR spectrum in order to correlate chemical species with the measured textures. One potential application is in characterizing inflammation, as the water signal of healthy tissue may be relatively organized compared to inflamed tissue, in which the water may have migrated, resulting in a more disordered composition.

## 3. Material and methods

**Figure 2.**
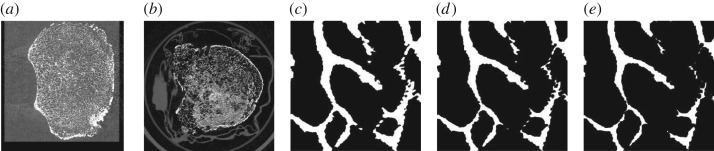
Example raw micro-CT image slices and comparison of thresholded and eroded images. (*a*) Example 9 μm-resolution micro-CT image slice from vertebral body dataset F60L3. The image slice lies in the transverse plane and is 56.1 mm wide and 59.4 mm long. (*b*) Example transverse slice from osteoporotic vertebral dataset AE15TH11, 52.0 × 52.0 mm. Image contrast has been increased in (*a*) and (*b*) to improve visibility. (*c*–*e*) A 5 × 5 mm section of micro-CT image, eroded at various stages to simulate effect of osteoporosis. (*c*) Baseline thresholded (healthy) section. (*d*) The same section as in (*c*), eroded with radius of 2 voxels (osteopenic). (*e*) The same section as in (*c*), eroded with radius of 4 voxels (osteoporotic).

To computationally validate the effectiveness of μTexture, and to identify optimal cross-section sizes and other measurement parameters for diagnostic power, we simulate μTexture data acquisition using test datasets constructed from micro-CT scans of *ex vivo* vertebral bone samples. We transform the micro-CT scans into frequency space and extract the signal intensities at frequencies relevant to trabecular bone texture. We first simulate μTexture measurements on the trabecular bone within two healthy vertebral bodies, labelled AE12L2 and F60L3. An example of a micro-CT image slice from vertebral body F60L3 is shown in figure 2*a*. We also simulate osteoporotic bone by artificially eroding the healthy bone images; examples of eroded regions at different levels of erosion are compared with a baseline thresholded image in figure 2*c*–*e*. Furthermore, we compare our results with those from two osteoporotic vertebral bodies, labelled AE15TH10 and AE15TH11; an example image slice from AE15TH11 is shown in figure 2*b*.

### 3.1. Micro-computed tomography image sets

We simulate μTexture measurements on micro-CT images of *ex vivo* human vertebral specimens obtained from a local organ bank and scanned at the Cartilage Tissue Engineering Lab (CTE) at the University of California, San Diego (UCSD). There are two image sets generated from two specimens from two non-osteoporotic patients, and two from an osteoporotic patient. Each set comprises image slices of one vertebral body. The specimens were obtained from different vertebrae; the non-osteoporotic image sets were taken from L2 and L3, and the osteoporotic image sets from TH10 and TH11. The non-osteoporotic vertebral bodies were obtained from a 75-year-old female (F60L3) and a 32-year-old male (AE12L2); neither patient had any bone-related diseases at the time of death. The two osteoporotic vertebral bodies were obtained from a 52-year-old male who died of chronic obstructive pulmonary disease and labelled AE15TH10 and AE15TH11. All images have a voxel size of 9 μm isotropic.

### 3.2. Sample preparation and imaging

The vertebral specimens are kept frozen before digestion with KOH. Specimens are thawed, and the vertebral bodies are dissected from the spinal column with a bone saw. Each vertebral body is placed in a beaker, to which 300 ml 1M KOH is added. The healthy samples are incubated at 56°C for 5 h, with the KOH replaced after the first 1.5 h of incubation. The healthy samples are washed with Milli-Q water several times to remove soft tissue, then incubated for another hour in KOH, for a total of 6 h of incubation. The osteoporotic samples are incubated for a total of only 4.5 h.

For all samples, the KOH is neutralized with the addition of glacial acetic acid at 0.052 times the volume of KOH. The samples are then washed with Milli-Q water, sonicated for 15 min at room temperature, washed again with Milli-Q water, then stored in 70% ethanol at room temperature before imaging. The samples are imaged in the sagittal plane using a Skyscan 1076 (Bruker, Kontich, Belgium) micro-CT scanner at a 9 μm voxel size.

### 3.3. Histomorphometry of trabecular bone

Histomorphometric analysis of micro-CT images is accomplished using a Bruker CT-analyser, or CTAn [24]. All images must be thresholded before histomorphometric analysis can be performed. The images are thresholded with 2D Otsu thresholding [25], followed by a ‘despeckling’ process in which black and white speckles, which are artefacts of image noise, below a specified threshold size are removed.

Analysis performed in the CTAn gives average Tb.Th and Tb.Sp values for dataset AE12L2 of 0.15 mm and 0.71 mm, respectively. F60L3 has slightly higher Tb.Th and Tb.Sp of 0.19 mm and 0.81 mm, respectively. While the sample is non-osteoporotic, it is taken from a considerably older patient. The Tb.Th values fall within ranges reported in the literature for human vertebral bone, though the Tb.Sp is slightly low [14,26].

AE15TH10 and AE15TH11, in comparison, have lower Tb.Th values, but also lower Tb.Sp values. The average Tb.Th for AE15TH10 and AE15TH11 is 0.072 and 0.073 mm, respectively, while the average Tb.Sp is 0.45 and 0.44 mm, respectively. However, these two datasets exhibit lower bone volume fractions of 10.7% and 10.0%, compared to 15.7% and 16.5% for AE12L2 and F60L3, respectively.

### 3.4. Volumes of interest selection

To focus the analysis on trabecular structure, we select VOIs from the interior trabecular region of the bone images, excluding portions of the images that contain the cortical shell or areas outside the bone. We subdivide this interior trabecular region into non-overlapping contiguous rectangular VOIs that encompass as much of the region as possible. From vertebral body F60L3, we generate a total of 106 5 × 5 × 5 mm VOIs from a usable region of trabecular bone spanning roughly 25 × 30 × 36 mm, and from vertebral body AE12L2, we generate a total of 166 (5 mm)^3^ VOIs from a region spanning roughly 30 × 30 × 35 mm. The size of the VOIs was chosen such that the superior–inferior, anterior–posterior and medial–lateral directions could be used as analysis directions, and where the VOI would be long enough in the analysis direction to contain several repeats of the trabecular pattern in order to achieve high signal-to-noise ratio. In calculating the ratio metric, we further subdivide the VOIs into 25 1 × 1 × 5 mm prisms, the signals from which are averaged together, as integrated power within our chosen frequency bands increases for narrower cross-sections (electronic supplementary material, figures S1 and S2). The same procedure is followed for the osteoporotic samples, yielding 13 VOIs for AE15TH10 and 15 VOIs for AE15TH11.

### 3.5. Image erosion

To simulate diseased bone, we artificially erode the micro-CT images of the healthy samples at various degrees to produce thinner trabeculae and wider spacings (figure 2*c*–*e*). The erosion process is performed by initially thresholding the images, following the Otsu and despeckling procedures described above, and then eroding the thresholded image with a kernel (or structuring element) of a chosen erosion radius. That is, a cubical (as the erosion is performed in 3D) kernel twice the erosion radius in length is used to remove voxels from the surfaces of each bone element in the VOI. The higher the erosion radius, the more voxels are removed (the thinner the bone elements). Image erosion is not performed on images of the osteoporotic samples.

**Figure 3.**
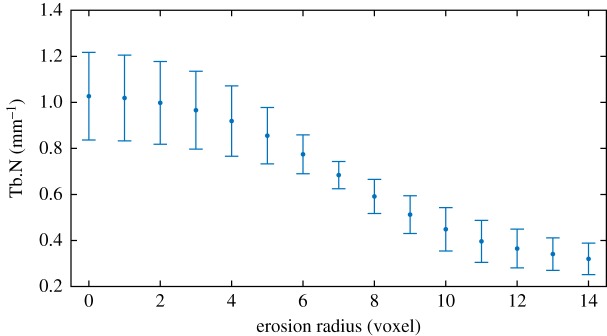
Trabecular number (Tb.N), which loosely represents the ‘frequency’ of the repeating trabecula-void pattern. 5 × 5 × 5 mm VOIs are thresholded, then eroded with varying erosion radii. One voxel corresponds to a (9 μm)^3^ cube. Error bars indicate one standard deviation from average over 10 samples.

Note that at higher erosion radii, such as in figure 2*e*, the trabecular elements can be eroded to the point of splitting in two, uniting gaps on either side of the elements. This can also result in isolated trabecular elements artificially created from the erosion process, though these are typically small enough to be identified and removed through the despeckling procedure. We also find that trabecular number decreases with increased erosion radius (figure 3).

## 4. Results

### 4.1. μTexture measurement simulation

Simulated μTexture measurements on human vertebral trabecular bone consist of intensities at specified frequency values within chosen VOIs. First, we select rectangular prisms from several stacked micro-CT image slices and collapse each prism in two chosen cross-section dimensions (i.e. averaging the 3D spatial signal in the two cross-section dimensions) to obtain a 1D spatial signal the length of the analysis dimension. We then compute the discrete Fourier transform of the 1D signal. However, a μTexture measurement examines one spatial frequency in one TR, though measurements at different spatial frequencies (as many as approx. 10 in a single excitation) are possible. Thus, to simulate a suite of μTexture measurements, we extract from the full spectrum the intensities of a selected subset of *k*-space values to represent a direct acquisition of signal intensities.

In order to obtain a Fourier spectrum that contains information regarding the texture of the trabecular bone, the length of the analysis dimension should be long enough to contain several repeats of the ‘pattern’ of trabecular bone and spacing. Furthermore, the prism should be relatively narrow in the cross-section dimensions, such that averaging over these dimensions does not result in excessive washing-out of structure. In practice, however, narrowing the cross-section size, while helpful in delineating structure, will also reduce the signal-to-noise ratio.

### 4.2. Trabecular ratio metric

Architectural parameters can be readily calculated from 2D images of trabecular bone with histomorphometry software. However, extracting structural information from frequency-domain data within a small subset of *k*-space is more subtle, particularly due to the variability in Tb.Th and Tb.Sp. We identify a quantity that can be extracted from a small number of *k*-values, as determined from simulated μTexture measurements, which can give insight into trabecular structure and serve as a diagnostic marker of bone disease.

Following Faber *et al.* [27,28], we calculate a Fourier transform ratio metric to characterize trabecular bone and classify baseline (healthy) and eroded (simulated osteoporotic) structures. Faber *et al.* began with 2D micro-CT images of trabecular bone. For each image, the discrete 1D Fourier transform was calculated line-by-line for each pixel row of the image. The Fourier spectra were then averaged, resulting in one 1D Fourier spectrum for each image. The ratio metric was then determined by averaging the intensities within a chosen low-frequency band and a chosen high-frequency band, taking the ratio of the average low-frequency intensity to the average high-frequency intensity, and taking the base-10 logarithm.

**Figure 4.**
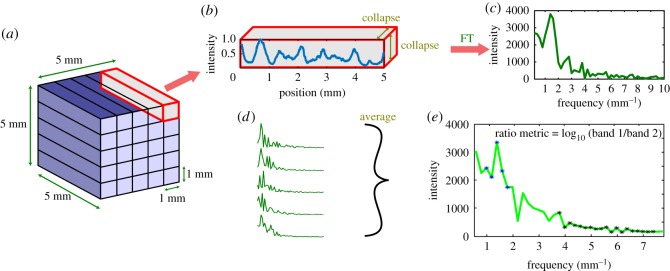
Workflow for calculating ratio metric. (*a*) A (5 mm)^3^ VOI is evenly divided into 25 1 × 1 × 5 mm prisms. (*b*) Each prism is collapsed along the dimensions measuring 1 mm in length. The resulting 1D signal in the spatial domain, plotted as a function of the length of the analysis dimension (5 mm), is shown normalized to between 0 and 1. (*c*) The Fourier transform is applied to the 1D (non-normalized) spatial signal. The full spectrum is truncated to show the first few peaks, excluding the DC signal. (*d*) 1D Fourier spectra are calculated in the same manner as (*b*) and (*c*) for all the prisms in (*a*), and averaged. (*e*) The ratio metric is calculated from the mean spectrum by averaging the points in the low-frequency band (blue stars) and averaging the points in the high-frequency band (black stars), taking the ratio of the two values, and computing the base-10 logarithm.

In contrast with the micro-CT images used in Faber *et al.*, μTexture is not used to obtain entire 2D position-space images. Hence, we develop an alternative method of calculating the ratio metric that more accurately simulates the μTexture measurement. First, we begin with 3D VOIs selected from the trabecular interior of the vertebrae. We then take various small samples within a VOI (figure 4*a*). Each sample is the same length as the original VOI in a chosen analysis direction, but is much narrower in the two cross-section directions. We find that the integrated power within our chosen frequency bands increases as the analysis length increases and the cross-section size decreases (electronic supplementary material, figures S1 and S2). We select these samples to have a cross-section size of 1 mm × 1 mm, which is on the order of the smallest resolution and machine parameters that can be acquired with μTexture, with the same analysis length of 5 mm. As in practice, the samples cannot overlap in position space, we choose the samples to lie side-by-side spanning the entire VOI, for a total of 25 non-overlapping samples, comparable to the number of samples that can be acquired in a few TRs with interleaved acquisition.

We then average each of these samples into a 1D signal along the analysis direction (figure 4*b*). We compute the discrete Fourier transform of each of these samples (figure 4*c*), average the spectra (figure 4*d*) and calculate the ratio metric (figure 4*e*). As in Faber *et al.* [27], the ratio metric is defined as the base-10 logarithm of the ratio of average signal intensity in the low-frequency band to the average intensity in the high-frequency band.

**Figure 5.**
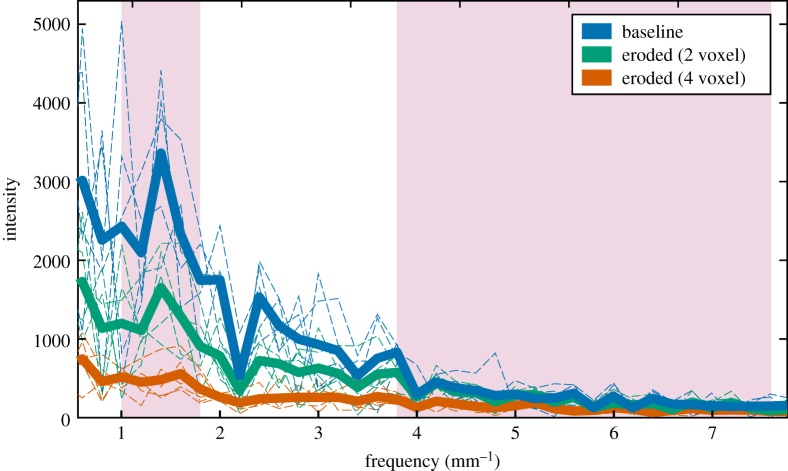
Comparing frequency-space intensities of healthy and simulated diseased bone. The Fourier spectra of a baseline thresholded VOI and the same VOI eroded to two different extents (2-voxel radius, simulating osteopenia; and 4-voxel radius, simulating osteoporosis) are shown. The baseline Fourier spectrum is the same as that shown in figure 4*e*. Each individual dotted line is generated by isolating a 1 × 1 × 5 mm prism generated from stacking micro-CT images, collapsing it in the cross-section (1 × 1 mm) dimensions, then taking the Fourier transform of the 5-mm-long 1D spatial signal. The thick lines are generated by averaging together the dotted lines of corresponding colour, such that each thick line represents a (5 mm)^3^ VOI. The pink shaded areas correspond to the low- and high-frequency bands used in calculating the ratio metric (figure 4).

In Faber *et al.* [27], the frequency bands corresponded to short and wide Tb.Sp ranges. In our case, we fix the band widths and locations to coincide roughly with Tb.Sp and Tb.Th distributions (of non-eroded structure) as determined with histomorphometric analysis in CTAn. The bands are located at [1, 1.8] mm^−1^ and [3.8, 7.4] mm^−1^ for low and high frequencies, respectively, corresponding to wavelengths of [0.56, 1] mm for Tb.Sp and [0.13, 0.26] mm for Tb.Th. However, the ratio metric is calculated from simulated raw μTexture measurements, which represent signal intensities, and as such actual values of Tb.Sp and Tb.Th are not used. Figure 5 compares sample Fourier transforms for a baseline thresholded VOI and the same VOI eroded to two extents to simulate osteopenia (2-voxel erosion radius) and osteoporosis (4-voxel radius). The frequency bands used to calculate the ratio metric are highlighted.

**Figure 6.**
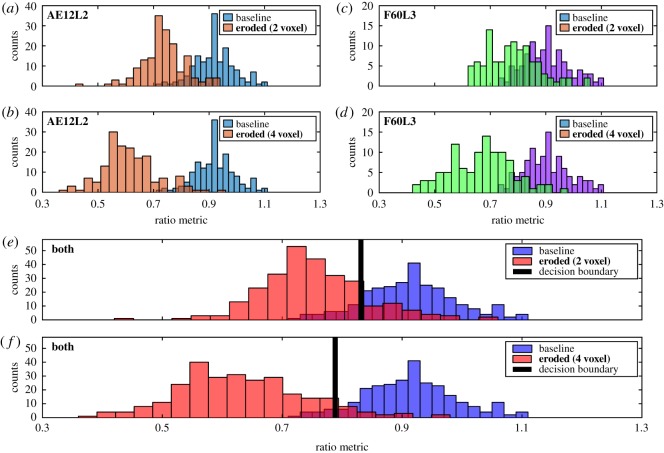
Comparing baseline and simulated diseased bone for the two healthy vertebral body samples. Each plot compares the histograms of ratio metric, a quantity determined from simulated μTexture measurements, for baseline data and simulated osteopenic or osteoporotic data, taken from a healthy vertebral dataset or datasets ((*a*,*b*) AE12L2; (*c*,*d*) F60L3; (*e*,*f*) AE12L2 and F60L3 combined). Each histogram (bars of the same colour) corresponds to the distribution of ratio metric values calculated from either the baseline (healthy) VOIs taken from a dataset, or the same VOIs after undergoing the erosion procedure to simulate osteopenic (2-voxel radius; *a*,*c*,*e*) or osteoporotic (4-voxel radius; *b*,*d*,*f*) damage. The ratio metric values were calculated by simulating μTexture measurements on 1 × 1 × 5 mm with a 5-mm medial–lateral analysis direction. A support vector machine (SVM) classifier is trained on the baseline and eroded ratio metric values plotted in (*e*) and (*f*) to determine the decision boundary (vertical black line) that best separates the two classes.

Figure 6 compares the distributions of ratio metric for baseline data and eroded data, for both the 2-voxel radius (simulated osteopenia) and 4-voxel radius (simulated osteoporosis) cases from vertebral image sets AE12L2 and F60L3. The respective baseline distributions for each dataset coincide with each other. The 2-voxel eroded distributions (figure 6*a*,*c*,*e*) overlap more strongly with the baseline distributions than the 4-voxel distributions (figure 6*b*,*d*,*f*), as is expected. The eroded distributions for F60L3 (figure 6*c*,*d*) are shifted to slightly higher values of ratio metric than for AE12L2 (figure 6*a*,*b*). Overall, the baseline and eroded distributions for both datasets remain mostly separate, but demonstrate some overlap between [0.7, 1.0].

We verify that a bone VOI can be correctly labelled as healthy or osteopenic/osteoporotic (eroded) based on the ratio metric. That is, we use the ratio metric as the sole input feature for two-class classification. We determine the decision boundary using a support vector machine (SVM) with a linear kernel function, implemented using MATLAB (The MathWorks Inc., Natick, MA, USA). We perform fivefold cross-validation and calculate the average sensitivity (fraction of eroded bone correctly classified) and average specificity (fraction of healthy bone correctly classified) to assess the classifier; we repeat this process a total of 50 times to minimize the effect of the partitioning of the data on the classification accuracy. Overall, we find that the sensitivity and specificity can vary significantly depending on the chosen analysis direction. For a classifier trained and tested on VOIs taken from dataset AE12L2, choosing the anterior–posterior analysis direction gave the highest sensitivity and specificity of 0.968 ± 0.003 and 0.954 ± 0.004, respectively, for the simulated osteoporotic (4-voxel eroded) case. For the simulated osteopenic (2-voxel eroded) case, the sensitivity and specificity are slightly lower due to the increased overlap in distributions and are 0.924 ± 0.008 and 0.916 ± 0.003, respectively.

For dataset F60L3 in the simulated osteoporotic case, the anterior–posterior analysis direction gave a sensitivity of 0.890 ± 0.009 and a specificity of 0.857 ± 0.005, while the medial–lateral analysis direction gave a slightly lower sensitivity of 0.888 ± 0.009 and a higher specificity of 0.907 ± 0.007. For simulated osteopenia, the anterior–posterior direction gave sensitivity and specificity of 0.762 ± 0.014 and 0.782 ± 0.012, respectively; the medial–lateral direction gave sensitivity and specificity of 0.761 ± 0.011 and 0.793 ± 0.014, respectively.

For a classifier trained and tested on VOIs from both healthy vertebral datasets combined, the medial–lateral direction gave the highest sensitivity (0.920 ± 0.003 for the 4-voxel case, 0.847 ± 0.004 for the 2-voxel case) and specificity (0.946 ± 0.003 for the 4-voxel case, 0.873 ± 0.006 for the 2-voxel case). The corresponding data and decision boundary are shown in figure 6*e*,*f*. Sensitivities and specificities, as well as the average ratio metric values, for each analysis direction and dataset are tabulated in the electronic supplementary material, tables S1 (for the 2-voxel case) and S2 (for the 4-voxel case). Moreover, for a diagnostic application, the decision boundary could be moved in order to prioritize minimizing false negatives, for example, at the expense of increasing the number of false positives.

**Figure 7.**
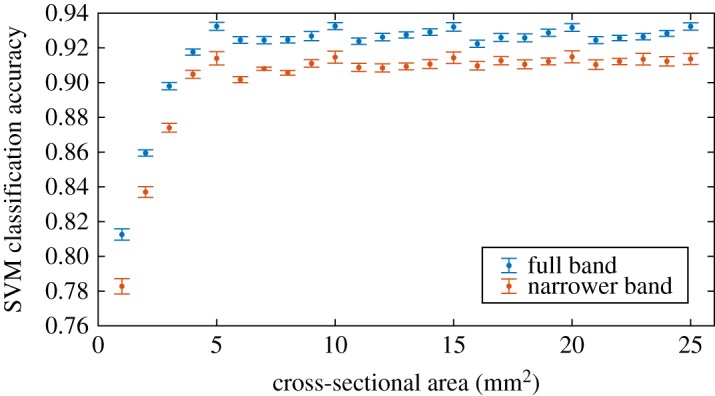
SVM classification accuracy for healthy and 4-voxel eroded bone as a function of cross-sectional area. The number of sub-VOI samples used to calculate the ratio metric is varied between 1 and 25, corresponding to cross-sectional area between 1 and 25 mm^2^. The accuracy does not exhibit a significant trend when the cross-sectional area is larger than 5 mm^2^. Training data are taken from both vertebral bodies AE12L2 and F60L3, with medial–lateral analysis direction. Blue points indicate ratio metric determined with original frequency bands; orange points indicate ratio metric determined with narrower high-frequency band (10 frequency points). Each point is averaged over 50 iterations of fivefold cross-validation to minimize partitioning bias; error bars indicate one standard deviation.

A question arises as to whether a smaller region of bone can provide sufficient diagnostic information. We determine the SVM classification accuracy when varying the number of sub-samples within each (5 mm)^3^ VOI, i.e. varying the size of the cross-sectional area of the prism targeted by μTexture. We systematically increase the number of sub-samples between one (a 1 × 1 × 5 mm prism, and thus the smallest possible resolvable cross-section) and 25 (constituting the entire VOI). For classifying healthy and 4-voxel eroded bone, we found that the SVM accuracy is significantly lower when the cross-sectional area is less than 5 mm^2^ (figure 7). However, for larger areas, the accuracy exhibits no significant trend, and any small variation in the accuracy could be attributed to small variation in the bone itself.

The frequency bands used for calculating the ratio metric contain 5 frequency points (for the low-frequency band) and 19 frequency points (for the high-frequency band). We investigate whether narrower bands, which would correspond to fewer μTexture measurements, result in a significant decrease in classification accuracy. We keep the same low-frequency band, but use a narrower high-frequency band of [3.8, 5.6] mm^−1^, which contains 10 points, to calculate the ratio metric, with a medial–lateral analysis direction. We train the SVM classifier on these values of the ratio metric for the thresholded and 4-voxel eroded bone sets, and we achieve, averaged over 50 runs of fivefold cross-validation, average sensitivity of 0.890 ± 0.005 and specificity of 0.938 ± 0.005. Figure 7 illustrates the change in classification accuracy as a function of total sample cross-sectional area.

### 4.3. Osteoporotic bone

**Figure 8.**
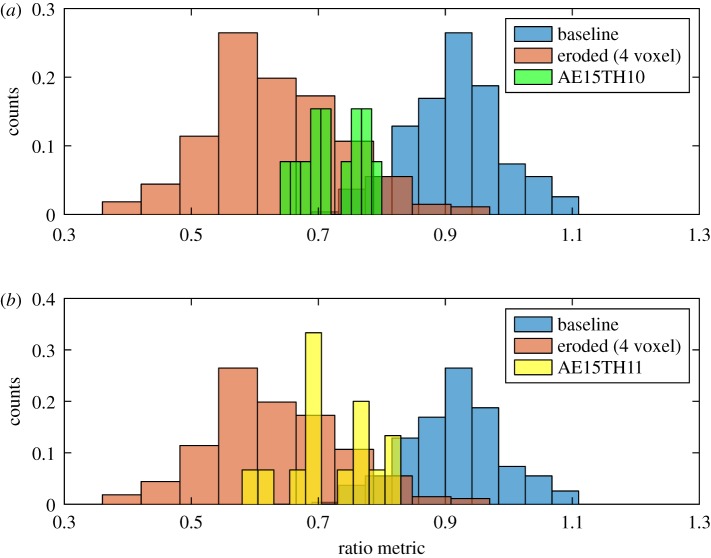
Comparing ratio metric distributions for healthy, eroded and osteoporotic bone. Each plot compares the ratio metric histograms for baseline (blue) and eroded (orange) VOIs from datasets AE12L2 and F60L3, and the ratio metric histogram for the thresholded VOIs from each of the osteoporotic datasets (AE15TH10, green, *a*; AE15TH11, yellow, *b*). As in figure 6, the ratio metric was calculated using the medial–lateral analysis direction. Owing to the much smaller number of osteoporotic VOIs, counts are normalized such that the total number of counts in each histogram equals 1.

We now apply our classifier trained on artificially eroded bone to images of bone with osteoporotic characteristics. The osteoporotic vertebral bodies AE15TH10 and AE15TH11 both contain a smaller volume of trabecular bone than the healthy bodies and thus we generate much fewer VOIs from the respective CT images, obtaining 13 (5 mm)^3^ VOIs from AE15TH10 and 15 VOIs from AE15TH11. We determine the ratio metric for each of the VOIs, and use the SVM classifier trained on the baseline and artificially 4-voxel eroded data (using VOIs from both healthy vertebral bodies) to classify the osteoporotic VOIs. We find that classification accuracy is higher for the medial–lateral analysis direction than the anterior–posterior analysis direction. Figure 8 compares the ratio metric distributions for AE15TH10 and AE15TH11 with the distributions from the healthy baseline and eroded data (from AE12L2 and F60L3, using the medial–lateral analysis direction) used to train the classifier. The osteoporotic ratio metric distributions coincide with the eroded ratio metric distribution, but also partially overlap with the baseline distribution. Applying the SVM classifier, VOIs from AE15TH10 are classified as osteoporotic with a sensitivity of 0.92, while VOIs from AE15TH11 are classified with a sensitivity of 0.80. Sensitivities and specificities for other analysis directions are tabulated in electronic supplementary material, table S3.

## 5. Discussion

We introduce an MR technique called μTexture, which can be used to rapidly acquire high-resolution information, at scales approximately 40 μm, about the complex architecture of biological tissues. Focusing on the specific case of osteoporosis in trabecular bone, we identify a diagnostic marker called the ratio metric that is predictive of deterioration in both osteoporotic and artificially eroded bone samples. Importantly, we demonstrate *in silico* that the ratio metric can be determined from only a few *k*-space values, which can be acquired rapidly with μTexture in small targeted regions within a bone. This procedure provides diagnostic information without the need to acquire an entire 2D MR image or even a 1D spectrum, thus avoiding the motion limitations that have previously limited the ability to probe complex bone architecture *in vivo*. By enabling the acquisition of predictive structural information in a short and non-invasive clinical procedure, μTexture has the potential to supplement traditional bone-density measurements and significantly improve the detection and monitoring of osteoporosis.

We demonstrate the feasibility of our proposed procedure through simulations of the μTexture measurement on high-resolution micro-CT bone data. We calculate the ratio metric from simulated measurements on healthy bone and artificially eroded versions of healthy bone, and find that an SVM classifier can distinguish the healthy and eroded bone using the ratio metric, with high sensitivity and specificity. We apply our classifier to simulated measurements of the ratio metric using micro-CT images of osteoporotic bone, and find that the metric is able to accurately classify healthy and diseased bone. We also show that a ratio metric measured with narrower frequency bands (i.e. fewer *k*-space measurements) can be used to classify healthy and eroded bone with only a minor sacrifice in accuracy, suggesting that μTexture measurements within only a few TRs could be sufficient to measure a diagnostic predictive of osteoporosis.

We note that a potential limitation of μTexture involves frequencies relevant to bone beyond which μTexture can probe. While the average trabecular thickness is on the order of 100 μm in healthy humans, Tb.Th for osteoporotic patients is much lower, and some trabeculae can be thinner than the approximately 40 μm limiting wavelength of μTexture. Despite this, our results in this paper show that μTexture is a promising tool for rapidly, non-invasively and effectively supplementing current methods of diagnosing and monitoring bone disease. Providing information about the complex architecture of bone, which is known to be a crucial factor in determining bone strength and fragility, this procedure has the potential to substantially improve osteoporosis detection.

We also note that characterization of bone strength depends not solely on the geometry and histomorphometry at this approximately 100 μm mesoscale, as can be probed with μTexture, but also on the micromechanics of bone constituents, such as mineralized collagen fibrils, at smaller scales [11,12]. μTexture is unable to probe these scales, but a combination of mesoscale textural measurements and microscale mechanical modelling can provide a more complete characterization of bone strength.

While this initial study focuses on trabecular bone, the methods described can be generalized to other biological tissues. μTexture can be used to investigate textural changes at scales down to approximately 40 μm (and smaller, with the use of machine learning techniques) in a variety of tissues, including the development of fibrosis in the lungs, liver, heart or kidney; the degradation of neuronal architecture with Alzheimer's and other neurodegenerative diseases; and the formation of tumours marked by angiogenesis, thereby informing diagnosis at early stages of disease. Furthermore, μTexture can be implemented clinically as a short, non-invasive procedure that can be repeated over time to monitor disease progression.

### 5.1. Methodological considerations

In this work, we introduce and perform an initial validation of the μTexture technique and the diagnostic ratio metric, using simulated measurements on a relatively small sample of four human vertebrae. Although these four bones provide a large set of VOIs for analysis and the results show promising classification performance, future work will examine the characteristics of trabecular bone across a larger dataset from a wider demographic range of individuals, in order to determine the performance of the proposed diagnostic across the population in clinical settings.

It is known that osteoporosis risk and bone architecture depend on several demographic characteristics. For example, women are more likely to develop osteoporosis than men, and the condition affects white, Hispanic and Asian women more than black women [29]. Future studies will characterize distributions of the ratio metric across a representative sample of the population, and determine how classification boundary depends upon factors such as age, ethnicity or sex.

In this analysis, we use artificial erosion of healthy bone samples as a model of bone disease, in addition to testing our methods on osteoporotic bone. This choice erodes all bone elements uniformly. However, this is not necessarily the case in actual osteoporotic bone tissue, especially due to preferential resorption of unloaded trabeculae. Indeed, we observe that variability and anisotropy are fundamental characteristics of trabecular bone architecture across the samples in this study. Previous studies have emphasized the relationship between the anisotropy of trabecular bone and its mechanical properties, though additional measures are needed to fully predict bone fracture [5,18]. The method proposed in this work determines the ratio metric through measurements on several small VOIs within the larger bone sample, without considering spatial variability in structure explicitly. Importantly, the method classifies healthy and diseased bone successfully even with this limitation. However, future work will extend the analysis of the variability in trabecular architecture in healthy and diseased bone. This variability in itself may provide important diagnostic information about the health and strength of trabecular bone, which could be leveraged to enhance the predictive capacity of the metric that we introduce here.

Finally, in this initial validation, we choose several parameters that may affect the classification outcome, including the sizes of the VOIs that μTexture samples from the bone. As described above, the ideal cross-sectional VOI size, across which the signal is averaged, will give a good trade-off between a higher signal-to-noise ratio and a finer structural resolution. For this analysis, we chose these sizes guided by both the practical limits on VOI size imposed by μTexture, and a study of which sizes produce the largest signal in the frequency bands of interest. Future work, however, will work to optimize this and other parameters through more in-depth investigations of larger datasets, in order to enable the best classification performance in clinical applications.

## Supplementary Material

Supplementary material: Figures S1 and S2; Tables S1, S2, and S3

## Data accessibility

The data reported in this paper are deposited at the following figshare repository: https://doi.org/10.6084/m9.figshare.5695399 [30].
