# Supplementary material for: Novel magnetic resonance technique for characterizing mesoscale structure of trabecular bone
Source: R Soc Open Sci. 2018 Aug 29;5(8):180563. doi: 10.1098/rsos.180563 (PMC6124118; doi:10.1098/rsos.180563)
Supplement: Supplementary material: Figures S1 and S2; Tables S1, S2, and S3 [file rsos180563supp1.pdf]

Supplementary material for Novel magnetic resonance technique for characterizing mesoscale structure of trabecular bone

Royal Society Open Science

Chantal Nguyen, Kimberly J. Schlesinger, Timothy W. James, Kristin M. James, Robert L. Sah, Koichi Masuda, and Jean M. Carlson

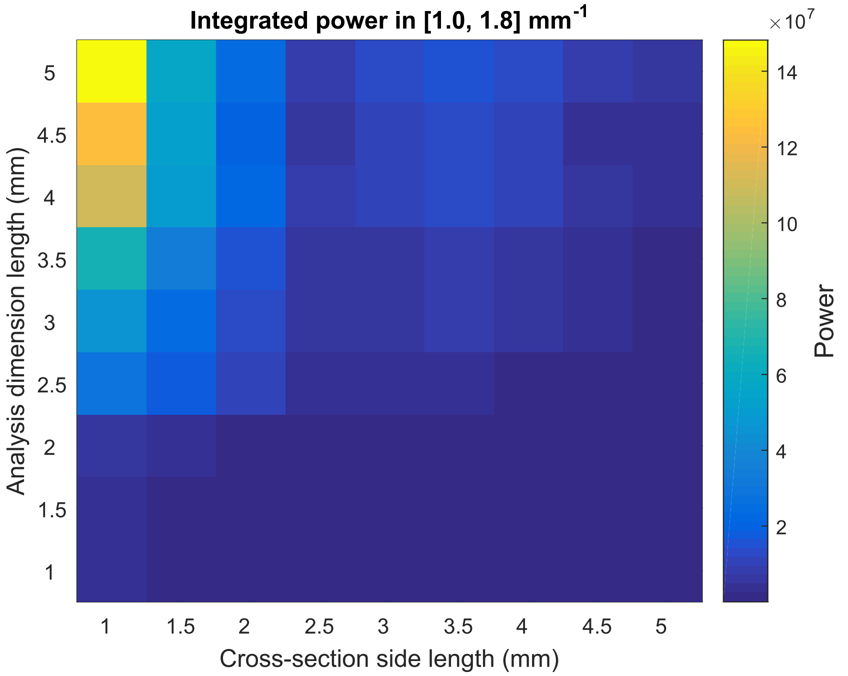

a

Figure S1: Integrated power increases as analysis length increases and cross-section size decreases. The integrated power in frequency band $[1.0, 1.8]$ mm^{-1}, corresponding to the low-frequency (Tb.Sp) band used to determine the ratio metric, is calculated for prisms of varying analysis dimension length and cross-section side length. The cross-section is kept square. Each data point is obtained by averaging over 100 prisms of the same size from different locations in the baseline thresholded AE12L2 dataset. The highest integrated power occurs for a prism with 1 mm $\times$ 1 mm cross-sectional area and 5 mm analysis length. To calculate the ratio metric, we use prisms of this size, which also corresponds to approximately the smallest resolution and machine parameters that can be acquired with µTexture.

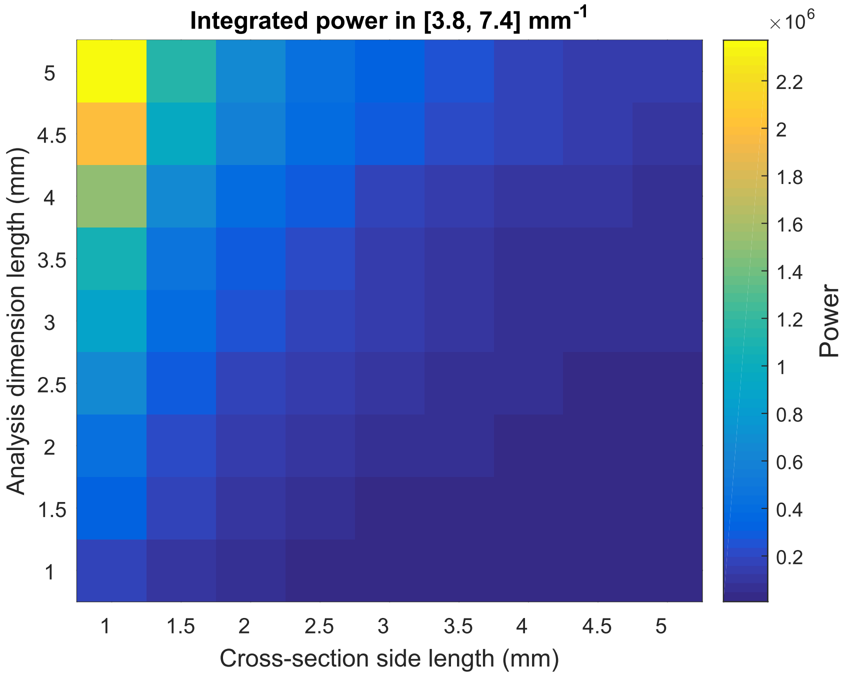

Figure S2: Integrated power increases as analysis length increases and cross-section size decreases. The integrated power in frequency band $[3.8, 7.4]$ mm^{-1}, corresponding to the high-frequency (Tb.Th) band used to determine the ratio metric, is calculated for prisms of varying analysis dimension length and cross-section side length. The cross-section is kept square. Each data point is obtained by averaging over 100 prisms of the same size from different locations in the baseline thresholded AE12L2 dataset. The highest integrated power occurs for a prism with 1 mm $\times$ 1 mm cross-sectional area and 5 mm analysis length. To calculate the ratio metric, we use prisms of this size, which also corresponds to approximately the smallest resolution and machine parameters that can be acquired with µTexture.

Table S1: Classification accuracy of thresholded and eroded (2-voxel radius) healthy bone samples.

Sample	Analysis direction	Mean ratio metric (baseline)	Mean ratio metric (eroded)	Sensitivity	Specificity
AE12L2	Anterior-posterior	0.95 ± 0.08	0.74 ± 0.08	0.924 ± 0.008	0.916 ± 0.003
AE12L2	Medial-lateral	0.92 ± 0.07	0.74 ± 0.08	0.890 ± 0.009	0.919 ± 0.004
AE12L2	Superior-inferior	0.91 ± 0.09	0.77 ± 0.10	0.761 ± 0.009	0.838 ± 0.007
F60L3	Anterior-posterior	0.95 ± 0.09	0.82 ± 0.10	0.762 ± 0.014	0.782 ± 0.012
F60L3	Medial-lateral	0.90 ± 0.08	0.78 ± 0.09	0.761 ± 0.011	0.793 ± 0.014
F60L3	Superior-inferior	0.72 ± 0.09	0.74 ± 0.11	0.433 ± 0.086	0.540 ± 0.087
Both	Anterior-posterior	0.95 ± 0.08	0.77 ± 0.10	0.841 ± 0.004	0.872 ± 0.004
Both	Medial-lateral	0.91 ± 0.08	0.75 ± 0.09	0.847 ± 0.004	0.873 ± 0.006
Both	Superior-inferior	0.84 ± 0.13	0.76 ± 0.10	0.549 ± 0.163	0.684 ± 0.101

Sensitivity (percentage of eroded samples correctly classified) and specificity (percentage of thresholded samples correctly classified) of classifiers trained on VOIs from indicated dataset(s) with specified analysis direction, as well as average ratio metric of baseline and eroded samples with 2-voxel radius (simulating osteopenia). Sensitivities and specificities are averaged over 50 runs of 5-fold cross-validation. Error denotes one standard deviation.

Table S2: Classification accuracy of thresholded and eroded (4-voxel radius) healthy bone samples.

Sample	Analysis direction	Mean ratio metric (baseline)	Mean ratio metric (eroded)	Sensitivity	Specificity
AE12L2	Anterior-posterior	0.95 ± 0.08	0.63 ± 0.09	0.968 ± 0.003	0.954 ± 0.004
AE12L2	Medial-lateral	0.92 ± 0.07	0.61 ± 0.10	0.927 ± 0.006	0.964 ± 0.002
AE12L2	Superior-inferior	0.91 ± 0.09	0.70 ± 0.11	0.858 ± 0.004	0.922 ± 0.006
F60L3	Anterior-posterior	0.95 ± 0.09	0.71 ± 0.11	0.890 ± 0.009	0.857 ± 0.005
F60L3	Medial-lateral	0.90 ± 0.08	0.67 ± 0.11	0.888 ± 0.009	0.907 ± 0.007
F60L3	Superior-inferior	0.72 ± 0.09	0.60 ± 0.11	0.656 ± 0.015	0.826 ± 0.015
Both	Anterior-posterior	0.95 ± 0.08	0.66 ± 0.10	0.920 ± 0.003	0.918 ± 0.004
Both	Medial-lateral	0.91 ± 0.08	0.63 ± 0.10	0.920 ± 0.003	0.946 ± 0.003
Both	Superior-inferior	0.84 ± 0.13	0.66 ± 0.12	0.801 ± 0.006	0.718 ± 0.004

Sensitivity (percentage of eroded samples correctly classified) and specificity (percentage of thresholded samples correctly classified) of classifiers trained on VOIs from indicated dataset(s) with specified analysis direction, as well as average ratio metric of baseline samples and eroded samples with 4-voxel radius (simulating severe osteoporosis). Sensitivities and specificities are averaged over 50 runs of 5-fold cross-validation. Error denotes one standard deviation.

Table S3: Classification accuracy of osteoporotic bone samples.

Sample	Analysis direction	Mean ratio metric	Sensitivity
AE15TH10	Anterior-posterior	0.74 ± 0.08	0.923
AE15TH10	Medial-lateral	0.72 ± 0.05	0.923
AE15TH10	Superior-inferior	0.75 ± 0.06	0.542 ± 0.027
AE15TH11	Anterior-posterior	0.79 ± 0.05	0.601 ± 0.009
AE15TH11	Medial-lateral	0.72 ± 0.07	0.800
AE15TH11	Superior-inferior	0.77 ± 0.09	0.327 ± 0.020

Average sensitivity for each set of osteoporotic VOIs classified using support vector machine classifier trained on baseline thresholded and 4-voxel eroded VOIs from AE12L2 and F60L3, as well as average ratio metric of osteoporotic samples. Sensitivities are averaged over 50 runs of 5-fold cross-validation. Error denotes one standard deviation.
